# EEG feature comparison and classification of simple and compound limb motor imagery

**DOI:** 10.1186/1743-0003-10-106

**Published:** 2013-10-12

**Authors:** Weibo Yi, Shuang Qiu, Hongzhi Qi, Lixin Zhang, Baikun Wan, Dong Ming

**Affiliations:** 1 Department of Biomedical Engineering, Tianjin University, Tianjin, China; 2 Tianjin Key Laboratory of Biomedical Detecting Techniques and Instruments, Tianjin, China

**Keywords:** Compound limb motor imagery, Event-related desynchronization, Event-related spectral perturbation, Power spectral entropy, Spatial distribution coefficient, Common spatial patterns, Support vector machine

## Abstract

**Background:**

Motor imagery can elicit brain oscillations in Rolandic mu rhythm and central beta rhythm, both originating in the sensorimotor cortex. In contrast with simple limb motor imagery, less work was reported about compound limb motor imagery which involves several parts of limbs. The goal of this study was to investigate the differences of the EEG patterns between simple limb motor imagery and compound limb motor imagery, and discuss the separability of multiple types of mental tasks.

**Methods:**

Ten subjects participated in the experiment involving three tasks of simple limb motor imagery (left hand, right hand, feet), three tasks of compound limb motor imagery (both hands, left hand combined with right foot, right hand combined with left foot) and rest state. Event-related spectral perturbation (ERSP), power spectral entropy (PSE) and spatial distribution coefficient were adopted to analyze these seven EEG patterns. Then three algorithms of modified multi-class common spatial patterns (CSP) were used for feature extraction and classification was implemented by support vector machine (SVM).

**Results:**

The induced event-related desynchronization (ERD) affects more components within both alpha and beta bands resulting in more broad ERD bands at electrode positions C3, Cz and C4 during left/right hand combined with contralateral foot imagery, whose PSE values are significant higher than that of simple limb motor imagery. From the topographical distribution, simultaneous imagination of upper limb and contralateral lower limb certainly contributes to the activation of more areas on cerebral cortex. Classification result shows that multi-class stationary Tikhonov regularized CSP (Multi-sTRCSP) outperforms other two multi-class CSP methods, with the highest accuracy of 84% and mean accuracy of 70%.

**Conclusions:**

The work implies that there exist the separable differences between simple limb motor imagery and compound limb motor imagery, which can be utilized to build a multimodal classification paradigm in motor imagery based brain-computer interface (BCI) systems.

## Background

Brain-Computer Interface (BCI) systems allow people to send messages or commands to an electronic device only by means of brain activity instead of muscular activity, and hence can provide an alternative communication and control channel for people with limited motor function to improve quality of their lives [1-4]. One kind of EEG-based BCI systems is based on the recording and classification of circumscribed and transient EEG changes in association with the imagination of different types of movements [5]. Motor imagery can modify the neuronal activity in the primary sensorimotor areas in a very similar way as observable with a real executed movement, so as a result it can serve to generate self-induced variations of the EEG [5,6]. Different from steady-state visual evoked potential (SSVEP) or event-related potential (ERP), self-induced brain activities could be interpreted as particular control signals which reflect subjective movement-related mental state of the user directly without any inducing factors outside.

Motor imagery may be seen as mental rehearsal of a motor act without any overt motor output [5]. Since Jasper and Penfield’s discovery of brain oscillatory activity induced by motor imagery [7], the development of brain-computer interface based on motor imagery has went through several decades. As early as 1996, the Graz BCI system was reported to discriminate between three simple limb motor imagery tasks (left hand, right hand, right foot), where band power estimations from three bipolar EEG channels were presented to a neuronal network-based classifier [8]. To confirm whether motor imagery could be available for patients with severe motor impairment, a tetraplegic patient had learned to operate an electrical driven hand orthosis by discrimination of two mental states to restore the hand grasp function [9]. On the other hand, motor imagery has already been applied as a brain switch in a hybrid BCI system by detecting the postimagery beta event-related synchronization (ERS) of foot movement imagination to turn on/off a four-step electrically driven hand orthosis with two flickering lights in order to reduce the false positive rate during resting period [10].

However, most research has been concentrated on to analyze the EEG rhythms induced by simple limb motor imagery involving single part of the limbs such as, e.g., left hand, right hand or foot. In recent years, less work was reported about brain oscillatory patterns induced by compound limb movement imagination. The characteristic intrinsic mode functions and brain synchrony between the supplementary motor area (SMA) and primary motor area (M1) have been studied during three kinds of motor imagination combining body with limb action [11]. Meanwhile, the limited numbers of classes contribute to the limited output commands, so for purpose of continuous three-dimensional control of a virtual helicopter in a three-dimensional space, both hands movement imagination was adopted to compensate the lack of instructions in simple limb motor imagery based BCI [12].

With respect to motor imagery of simple limb movement, several parts of limbs like hand (forearm, postbrachium) and foot (shank, thigh) are involved in compound limb movement imagination, which may activate the neurons oscillation in multiple functional areas of cerebral cortex and, at the same time, also can satisfy the requirements of multiple instructions output of control information in motor imagery based BCI systems. The research on compound limb motor imagery has great significance for the limb function rehabilitation for the patients suffering from severe motor injury.

In addition, for motor imagery based BCI system, a great variety of algorithms have been frequently used to extract EEG features of different mental states such as band power (BP) estimation, power spectral density (PSD) values and autoregressive (AR) [13-16]. However, it is very difficult to differentiate between more than two mental states when only imagery-induced ERD patterns are available [17]. CSP is an algorithm based on the simultaneous diagonalization of two matrices, which has been widely used in BCI systems as it is well suited to discriminate different MI patterns. The goal of CSP is to design a spatial filter that projects raw signals to new time series whose variances are optimal for the discrimination of two mental tasks, namely maximize the variance of bandpass filtered EEG signals from one class while minimizing their variance from the other class. Although the method of CSP has been applied to discriminate two movement-related patterns successfully [18-20], it is restricted to binary problems. Therefore, a one-versus-rest scheme has been applied to extend this algorithm to the multi-class case [21].

In this study, seven kinds of mental tasks have been designed, involving three tasks of simple limb motor imagery (left hand, right hand, feet), three tasks of compound limb motor imagery combining hand with hand/foot (both hands, left hand combined with right foot, right hand combined with left foot) and rest state. The goal of this paper is to investigate the differences of the induced brain oscillatory patterns between simple limb motor imagery and compound limb motor imagery by event-related spectral perturbation (ERSP), power spectral entropy (PSE) and spatial distribution coefficient. In order to verify the feasibility of the application of seven mental tasks to BCI systems, the CSP algorithm was used and extended to the multi-class case in a one-versus-rest scheme for seven-class feature extraction. Three kinds of multi-class CSP algorithms were applied and compared by the classification performance.

## Methods

### Experimental procedure

**Figure 1:**
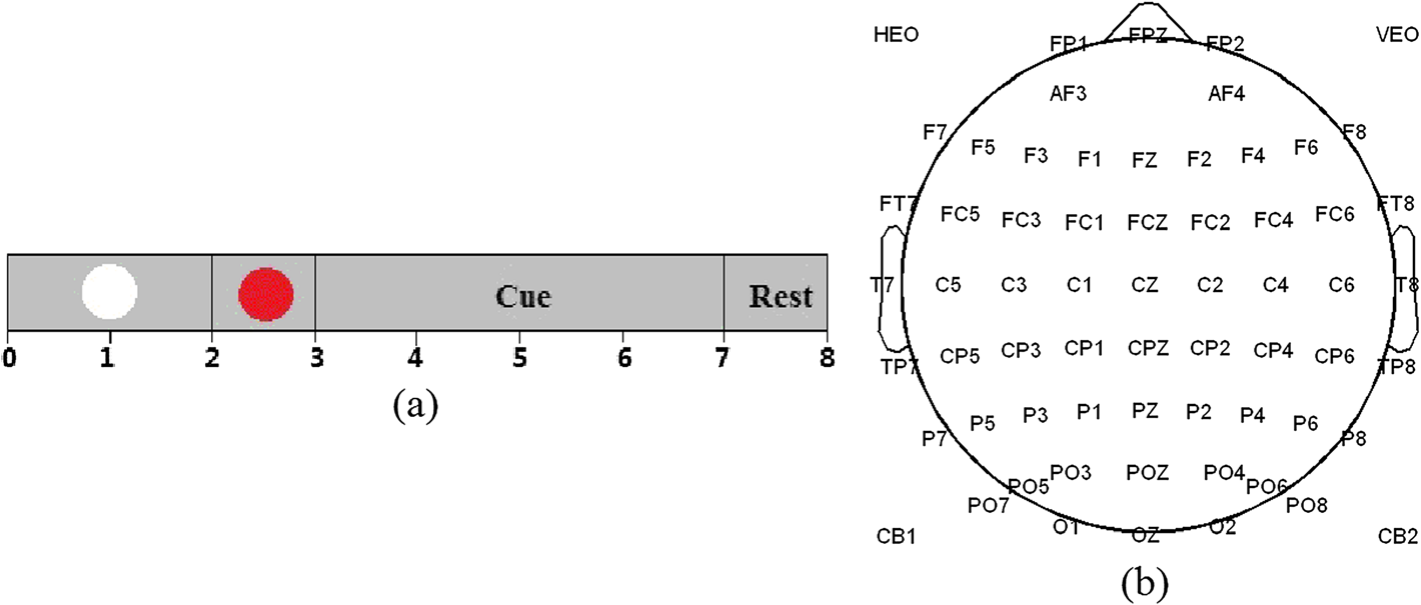
**Experimental paradigm and electrode positions. (a)** Experimental paradigm of one trial. **(b)** 64-electrode positions.

Ten right-handed healthy subjects (7 females and 3 males, 23–25 years old) participated in this experiment. All of the subjects have no prior experience with motor imagery based BCI before. They were required to take one week of training before EEG recording. The subjects were sitting in a chair at one-meter distance in front of a computer screen. Each trial (8 seconds) began with a white circle at the center of the monitor for 2 seconds. At second 2, a red circle (preparation cue) appeared on the screen to remind the subjects of paying attention to the character indication next. And then at second 3, red circle disappeared and character indication ('Left Hand’, 'Left Hand & Right Foot’, et al.) was presented on the screen for 4 seconds. The participants were asked to concentrate mind on performing the indicated motor imagery task kinesthetically rather than a visual type of imagery while avoiding any motion during imagination. At the end of imagination, 'Rest’ was presented for 1 second before next trail (Figure 1a). The experiments were divided into 9 sections, involving 8 sections consisting of 60 trials each for six kinds of motor imagination tasks (10 trials for each movement imagination in one section) and one section consisting of 80 trials for rest state. The sequence of six motor imagination tasks was randomized. There were breaks of 5 to 10 minutes between sections. So there are 560 trials (80 trials for each type of mental task) in the dataset totally for the following study.

EEG data was recorded from 64 Ag/AgCl scalp electrodes placed according to the International 10/20 System referenced to nose and grounded prefrontal lobe (Figure 1b). The EEG signals were acquired by a Neuroscan SynAmps2 amplifier whose sampling rate is 1000Hz and band-pass filtering range is 0.5-100 Hz. Besides, an additional 50-Hz notch filter was used during data acquisition. Thereafter, the original EEG signals were band-pass filtered between 1 and 40Hz, and then downsampled at 200Hz. Before further analysis, common average reference (CAR) was adopted here in the pre-processing.

The study was approved by the ethical committee of Tianjin University. All subjects signed informed consent in advance.

### Event-related spectral perturbation

The event-related spectral perturbation (ERSP) method allows us to observe the spectral power changes of the induced EEG relative to the stimulus from the views of time-frequency domain, which could supply more details about ERD/ERS patterns of different types of motor imagery. Changes of event-related spectral power were analyzed with ERSP defined as follows:

(1) $$\mathrm{ERSP}\left(f,t\right)=\frac{1}{n}\sum_{k=1}^{n}\left(F_{k}{\left(f,t\right)}^{2}\right)$$

where *n* is the number of trails, and *F*_*k*_*(f, t)*is the spectral estimation of *k*th trial at frequency *f* and time *t*[22]. Mean ERSP values were calculated from -3000ms to 5000ms and displayed between 1 and 35Hz for every mental task. In this study, the time-frequency ERD/ERS maps from three key electrode positions C3, Cz, C4 were presented for analysis.

To explore more clear information about the ERD band range, we averaged the ERSP values across the imagination period (4 s) in order to obtain the power changes of EEG with frequency for different mental tasks. Then the mean power changes were computed by averaging over all subjects. To verify the differences of ERD band range between simple limb motor imagery and compound limb motor imagery, we constructed six groups for comparison: BH VS LH, BH VS RH, LH&RF VS LH, RH&LF VS RH, LH&RF VS F, and RH&LF VS F.

In addition, topographical distribution is a method for us to figure out which areas of the brain are involved when ERD occurs during the imagination of different types of movements. Based on the ERSP values from 60 electrodes (except HEO,VEO, CB1 and CB2), the averaged ERSP value in the fixed frequency band and time interval within alpha band was calculated.

### Power spectral entropy and spatial distribution coefficient

Entropy provides a physical measurement to assess the order of a system [23]. Modified from Shannon’s definition of entropy, power spectral entropy (PSE) estimates the changes in the amplitude component of the power spectrum of the EEG, using the amplitude components at each frequency of the power spectrum as the probabilities in the entropy calculations [24]. It can be calculated as the following formula [25]:

(2) $$H=-\sum_{i=1}^{n}p_{i}\ln\left(p_{i}\right)$$

Where *Pi* is the value of power spectral density at each frequency point of the EEG signal, *n* is the number of frequency points. A high PSE implies a flat, uniform spectrum with a broad spectral content, while a low PSE implies a spectrum with all the power condensed into a single frequency bin [24]. In this paper, PSE value of each movement imagination was calculated from 5 to 35Hz and averaged over 80 trials. To verify the differences between simple limb motor imagery and compound limb motor imagery, we also constructed six groups for comparison as above.

On the other hand, spatial distribution coefficient was proposed to investigate the distinct between simple limb motor imagery and compound limb motor imagery from the view of spatial distribution. The spatial distribution coefficient can be calculated based on Equation (2), as follows:

(3) $$H_{\mathrm{sdc}}=-\sum_{i=1}^{m}q_{i}\ln\left(q_{i}\right)$$

where *m* denotes the number of channels. The input *q*_*i*_ is defined as follows:

(4) $$q_{i}=\frac{\left|m_{i}-r_{i}\right|}{r_{i}}$$

where *m*_*i*_ and *r*_*i*_ are the mean power spectral density of alpha band at each channel from imagination tasks and rest state respectively.

### CSP algorithms

Common Spatial Patterns algorithm is a method to extract features of two classes based on multi-channel EEG information [26]. In this study, the raw data was band-pass filtered between 8 Hz and 30 Hz [20], time interval starting from 3.5 s to 6.5 s. Since the property of CSP for binary situation, it has to be modified to be appropriate to the circumstance of multi-class MI tasks. We present here three multi-class CSP algorithms: multi-class CSP (Multi-CSP), multi-class CSP based on generalized eigenvector (Multi-GECSP), multi-class stationary Tikhonov regularized CSP (Multi-sTRCSP).

1) *Multi-CSP*

For the analysis, the multi-channel EEG data of a single trial is represented as an N*T matrix *X*_*i*_, where *i∈*{1,2,,7}, *N* is the number of channels and *T* is the number of samples per channel. Similar to the steps in [20], we firstly obtain average covariance matrix ∑_*i*_ of each MI pattern,*i∈*{1,2,,7}. The whitening matrix can be obtained by

(5) P=Λ-12U0T

Where *U*_*o*_ is the matrix of eigenvectors and ∧ is the diagonal matrix of eigenvalues from

(6) $$\text{Σ}=\sum_{i=1}^{7}{\text{Σ}}_{i}=U_{0}\Lambda U_{0}^{T}$$

Thereafter, in order to acquire the spatial filter matrix relevant to the first class, we let ${\text{Σ}}_{1}^{'}=\sum_{i=2}^{7}{\text{Σ}}_{i}$ according to the strategy of one-versus-rest, and if ∑_1_ and ${\text{Σ}}_{1}^{'}$ can be translated as

(7) $$\begin{array}{l} Y_{1}=P{\text{Σ}}_{1}P^{T}\\ Y_{1}^{'}=P{\text{Σ}}_{1}^{'}P^{T} \end{array}$$

Then *Y*_1_ and $Y_{1}^{'}$ share common eigenvectors

(8) $$\begin{array}{l} Y_{1}=U_{1}{\text{Λ}}_{1}U_{1}^{T}\\ Y_{1}^{'}=U_{1}{\text{Λ}}_{1}^{'}U_{1}^{T} \end{array}$$

With the projection matrix $W_{1}=U_{1}^{T}P$ consisting of spatial filters corresponding to the first class, the other six projection matrices also can be gained similarly.

2) *Multi-GECSP*

In this approach, formally, the calculation of projection matrix *W* can be solved by maximizing the Rayleigh quotient, as the following function [26-28]:

(9) $$R\left(W\right)=\frac{W^{T}{\text{Σ}}_{i}W}{W^{T}\text{Σ}W}$$

The maximization of the Rayleigh quotient can be reformulated as a constrained optimization problem, which can be solved using Lagrange multiple. The solution *W* satisfying the equation (8) can be achieved by solving the generalized eigenvalue problem:

(10) $${\text{Σ}}_{i}W=\lambda \left(\text{Σ}\right)W$$

where the generalized eigenvector with largest eigenvalue corresponds to the spatial filter matrix *W* that maximizes the variance of class *i* while minimizing the common variance [28].

3) *Multi-sTRCSP*

CSP is also known to be highly sensitive to noise and prone to overfitting [7,8], in order to overcome this problem, one proposed method based on Tikhonov regularization (TR) of the objective function is to add a penalty termP_TRCSP_ (*W*) = ∥*W*∥^2^ = *W*^*T*^*W* = *W*^*T*^*IW* in the denominator [27]. Otherwise, the sCSP aims at extracting robust and stationary features [28], where the penalty term *P*_*sCSP*_(*W*) can be obtained by minimizing the following function for each class *i*:

(11) $$D_{i}\left(W\right)=\sum_{k}\left|W^{T}{\text{Σ}}_{i}^{\left(k\right)}W-W^{T}{\text{Σ}}_{i}W\right|$$

where $\Sigma_{i}^{\left(k\right)}$ is the covariance matrix of the *k*th trial of class *i*.

Combining both approaches mentioned above together, the stationary Tikhonov regularized CSP (sTRCSP) maximizes the following objective function:

(12) $$R\left(W\right)=\frac{W^{T}{\text{Σ}}_{i}W}{W^{T}\text{Σ}W+\alpha P_{\mathrm{sCSP}}\left(W\right)+\beta P_{\mathrm{TRCSP}}\left(W\right)}$$

Where *a* and *β* are regularization parameter both chosen among {0, 2^-8^,2^-7^,2^-6^,2^-5^,2^-4^,2^-3^,2^-2^,2^-1^,2^0^} by tenfold cross-validation.

### Classification

Support vector machine (SVM) is a classical method for pattern recognition in BCI systems using the optimal discriminant hyperplane to identify classes, which is adopted here for classification of seven kinds of MI patterns. SVM is known to have good generalization properties, to be insensitive to overtraining and be suitable to small training sets [29,30]. In this study, we used LIBSVM software package [31], a freely-available library of SVM tools, to solve the multi-class classification problem. The original multi-channel (64) EEG data was preprocessed firstly (downsampled at 200Hz, common average referenced, band-pass filtered between 8Hz and 30Hz, time interval starting from 0.5s to 3.5s after cue). Then whole dataset was divided into a training set and a testing set. The training set served as the input of multi-class CSP algorithms in order to achieve CSP filters which were used to extract features. The classifier calculated based on the training set was used to classify the testing set. The estimation of the classification accuracy was executed by a tenfold cross-validation strategy, which means each portion S^(k)^will be used as the testing set once. *S*^(k)^ = {*s*_*i,j*_ ∣*s*∈*S,i*∈{1,2,,7},*j*∈*J*^(k)^} is the sample set of the *k*th fold, *K*∈{1,2,,9,10}. *S* donates the whole dataset while *i* is the type of mental task and *J*^*(k)*^donates the sequence number chosen for each mental task in *k*th fold. The final classification accuracy was then computed by averaging over all results of testing sets, $\mathrm{acc}=\frac{1}{10}\sum_{k=1}^{10}\mathrm{ac}c^{\left(k\right)}$where *a*cc^*(k)*^ is the accuracy for *k*th fold.

## Results

### EEG patterns during seven mental tasks

#### Event-related spectral perturbation

**Figure 2:**
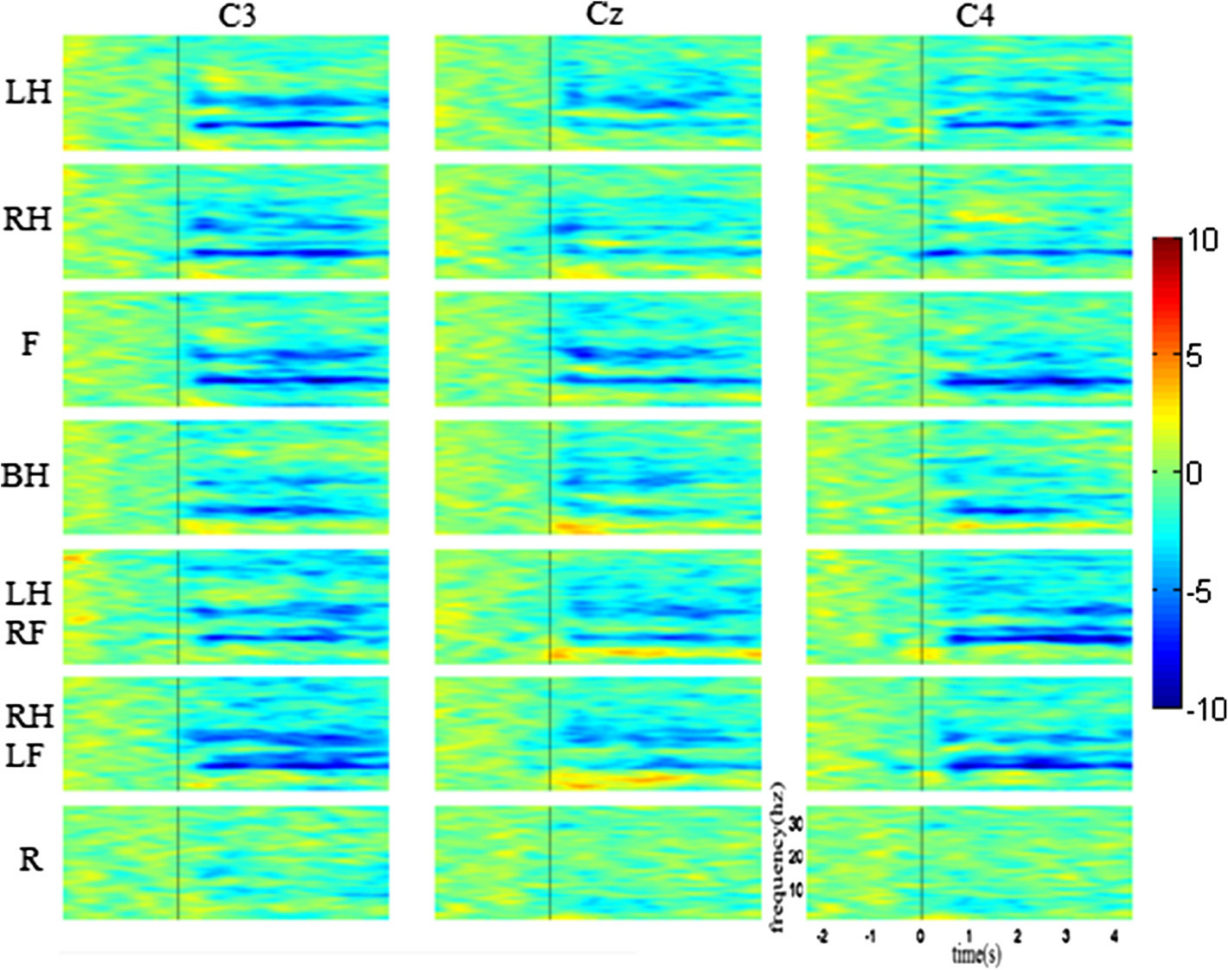
**Examples of time-frequency maps for one subject, 7 mental tasks, and 3 electrode locations.** LH, RH, F, BH, LH&RF, RH&LF and R indicate left hand, right hand, feet, both hands, left hand combined with right foot, right hand combined with left foot and rest respectively. Blue indicates ERD.

Figure 2 shows the time-frequency maps of seven kinds of MI tasks (blue indicates ERD) from one subject for electrode positions C3, Cz and C4. Here, left hand, right hand, feet, both hands, left hand combined with right foot, right hand combined with left foot and rest are represented by LH, RH, F, BH, LH&RF, RH&LF and R. The maps show obvious long-lasting power decrease in both alpha and beta rhythm starting almost 500 ms after stimulus onset for all motor imagery except rest. ERD patterns in 8-9Hz band can be found at C3 and C4 during both hands imagery, but there is no obvious difference between each other. Quite different patterns are found during compound limb motor imagery combining hand with contralateral foot. Compared with simple limb motor imagery (left hand, right hand, feet), the ERD feature bands of compound limb motor imagery overlap with that of the former one but more broad as well, especially in alpha rhythm (8-11Hz) at electrode positions C3 and C4 during left hand combined with right foot imagery and right hand combined with left foot imagery.

**Figure 3:**
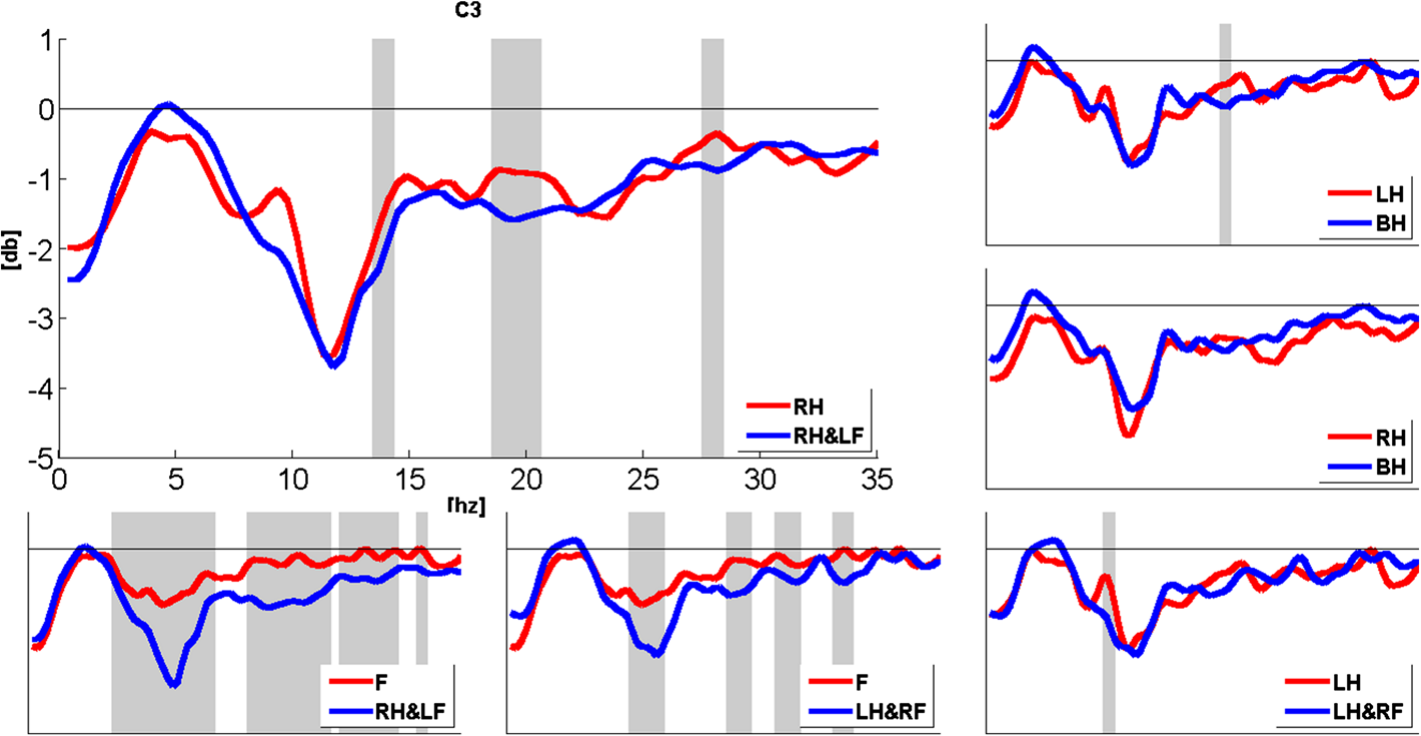
**The comparison of power changes in six groups for electrode position C3.** Blue line indicates compound limb motor imagery, while red line indicates simple limb motor imagery. The grey blocks present statistic significant differences (p < 0.05) between simple limb motor imagery and compound limb motor imagery.

**Figure 4:**
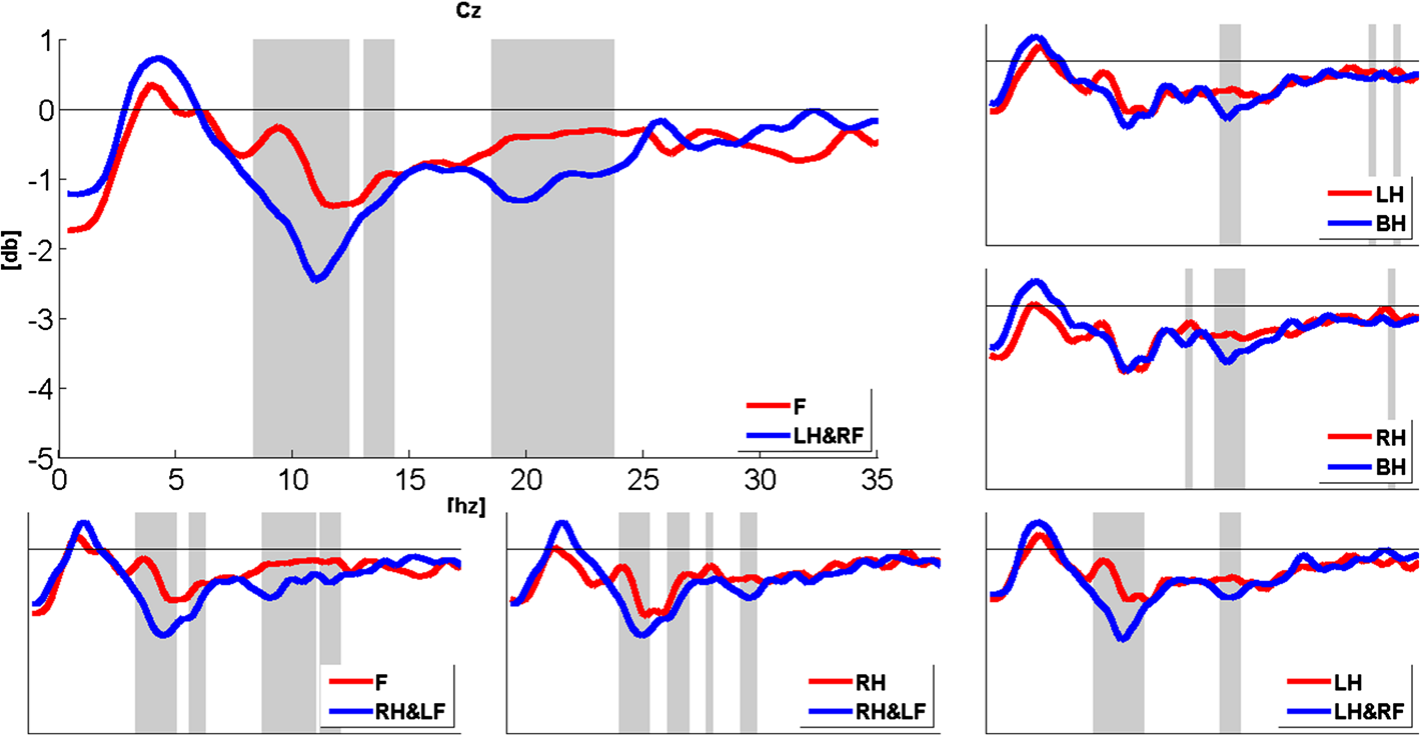
**The comparison of power changes in six groups for electrode position Cz.** Blue line indicates compound limb motor imagery, while red line indicates simple limb motor imagery. The grey blocks present statistic significant differences (p < 0.05) between simple limb motor imagery and compound limb motor imagery.

**Figure 5:**
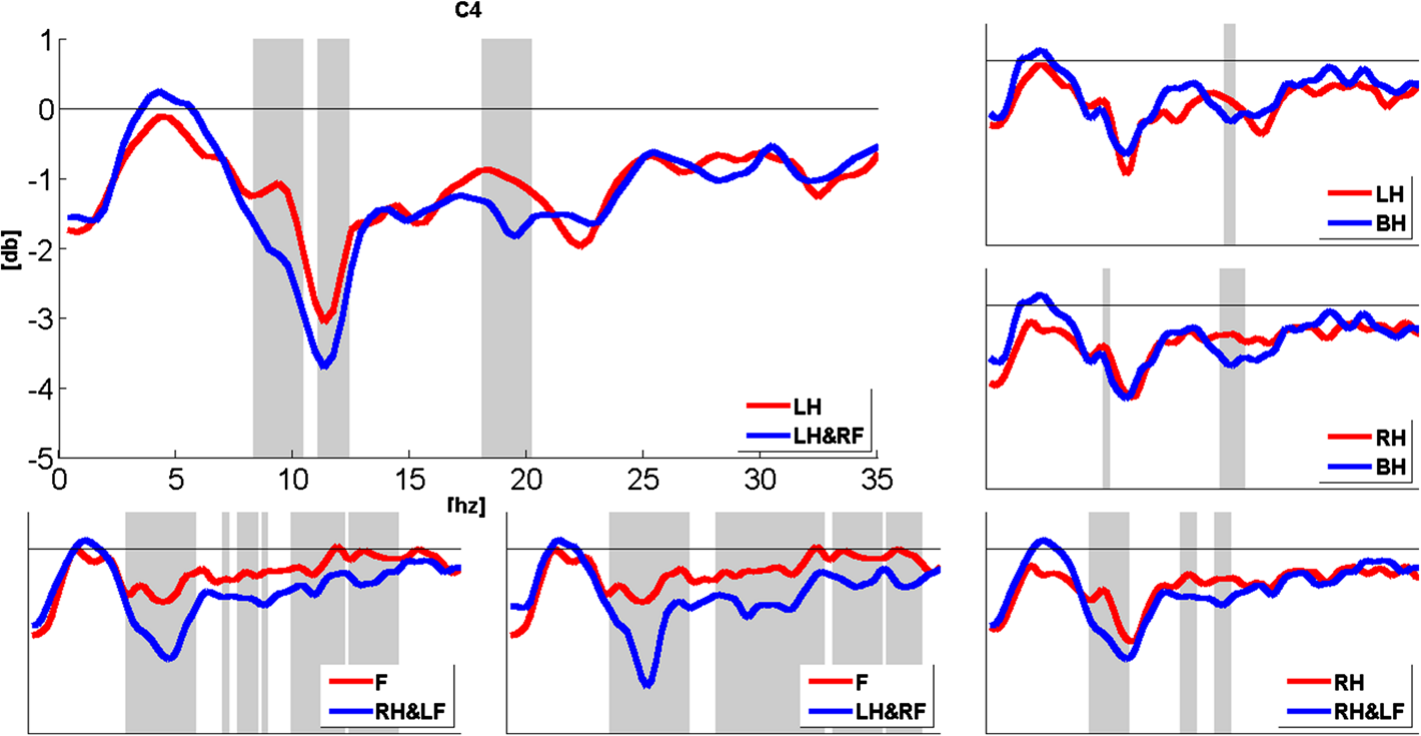
**The comparison of power changes in six groups for electrode position C4.** Blue line indicates compound limb motor imagery, while red line indicates simple limb motor imagery. The grey blocks present statistic significant differences (p < 0.05) between simple limb motor imagery and compound limb motor imagery.

To observe the expansion of ERD band during compound limb motor imagery more clearly, Figures 3, 4 and 5 show the comparison of power changes in six groups for electrode C3, Cz and C4. The paired *t*-test was used, and the significant differences (p < 0.05) between two conditions were shaded by grey blocks. From Figure 3, we can see clearly that the ERD band within alpha rhythm is broader than right hand imagery during right hand combined with left foot imagery at C3. In addition, significant differences are found around 14 Hz, 20Hz and 28Hz. Compared with feet imagery, significant differences appear within several sub-bands during left/right hand combined with contralateral foot imagery. From Figure 4, it also can be seen that compared with feet imagery, more broad-banded ERD occurs during left hand combined with right foot imagery at Cz, accompanied with significant differences within almost whole alpha band and 18-24Hz band. In other groups, there exist significant differences to a variable extent between compound limb motor imagery and simple limb motor imagery. Meanwhile, compared with left hand imagery, we can see from Figure 5 that besides the broader alpha-band ERD, there also exists a slightly 18-20Hz ERD with significant difference at C4 during left hand combined with right foot imagery. Furthermore, several broad-banded significant differences are observed between left/right hand combined with contralateral foot imagery and feet imagery. And there also exists a broader alpha-band ERD during right hand combined with left foot imagery.

**Figure 6:**
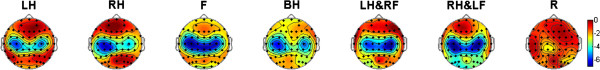
**The topographical distribution for 7 mental tasks from one subject.** The maps are made based on ERSP values of each electrode. Blue regions indicate the involved areas when ERD occurs during mental tasks.

Apart from the EEG signal analysis in time-frequency domain, spatial distribution analysis also plays an important role in exploring EEG patterns of different mental tasks. The topographical distributions of 7 mental tasks obtained from one subject are presented in Figure 6. It can be found that ERD of alpha band occurs on all central electrode positions during each motor imagination except rest, which means the spatial distribution of the induced ERD feature on brain surface mainly focus on the sensorimotor areas corresponding to human limbs. The ERD feature of both hands imagery appears on both left and right hand areas, which is different from the spatial distribution of single hand imagery, but the ERD is slightly weaker in right hemisphere as compared to left hemisphere. From the distribution during left/right hand combined with contralateral foot imagery, we can see the strong ERD on both hand areas and another midcentral ERD, which is obviously distinct from the distribution of motor imagery only involving hand (left hand, right hand, both hands).

#### Power spectral entropy

**Figure 7:**
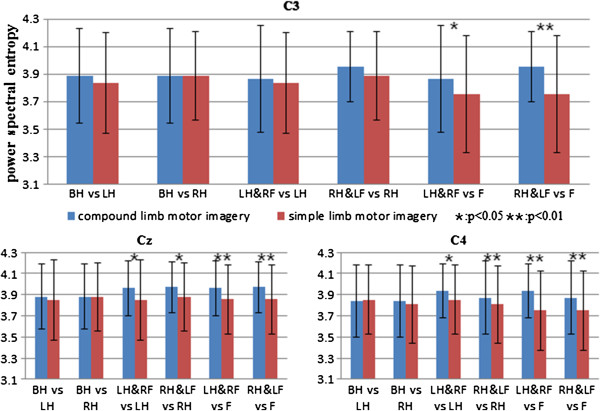
**The comparison of PSE values in six groups for C3, Cz and C4.** Blue bar indicates compound limb motor imagery, while red bar indicates simple limb motor imagery. Condition pairs that significantly differ from each other are indicated by an asterisk (p < 0.05) or two asterisks (p < 0.01).

Power spectral entropy is applied to better understand the phenomenon of ERD band expansion during compound limb motor imagery. Figure 7 gives the mean PSE values across ten subjects for three electrode positions. Blue bar indicates compound limb motor imagery, while red bar indicates simple limb motor imagery. It can be observed that most asterisks appear upon the last four groups, which indicates the existence of significant differences on the PSE values during compound limb motor imagery combining left/right hand with contralateral foot. The result shows the PSE values of left/right hand combined with contralateral foot imagery are significantly higher than that of feet imagery at electrode positions C3 (p = 0.015 and p = 0.009). In addition, not only the PSE value of left hand combined with contralateral foot imagery is significantly higher than that of left hand and feet imagery, but the PSE value of right hand combined with contralateral foot imagery is significantly higher than that of right hand and feet imagery as well at electrode positions Cz and C4.

#### Spatial distribution coefficient

**Figure 8:**
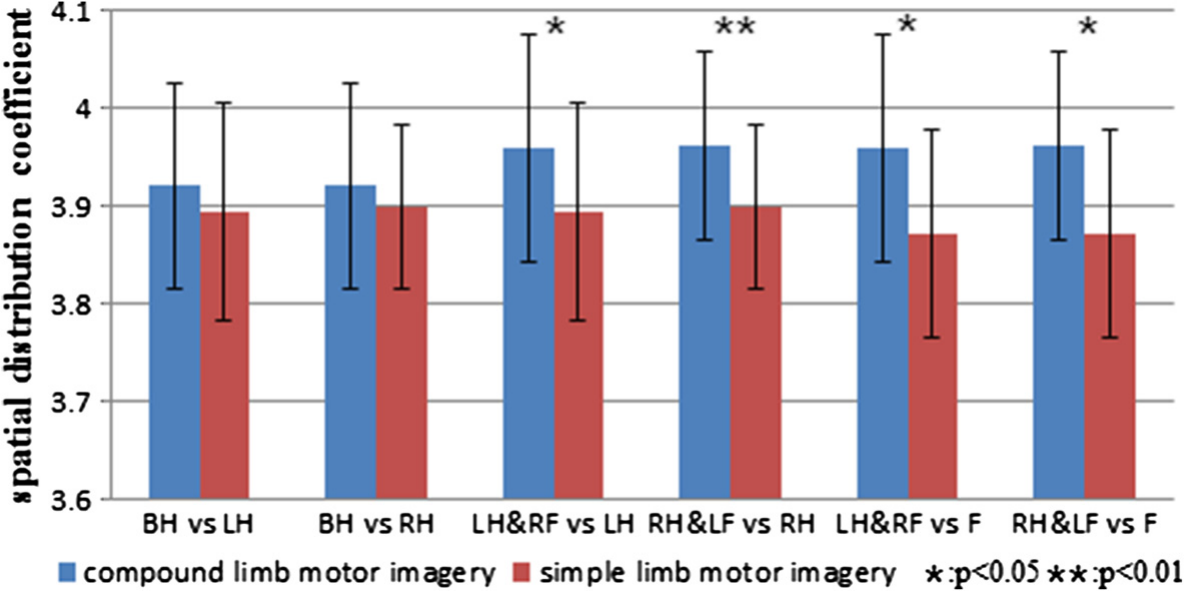
**The comparison of spatial distribution coefficient among six groups.** Blue bar indicates compound limb motor imagery, while red bar indicates simple limb motor imagery. Condition pairs that significantly differ from each other are indicated by an asterisk (p < 0.05) or two asterisks (p < 0.01).

Spatial distribution coefficient is introduced to quantify the difference in spatial distribution between simple limb motor imagery and compound limb motor imagery. Figure 8 shows the comparison of spatial distribution coefficient among six groups. The result presents existence of significant differences on the spatial distribution coefficients during compound limb motor imagery combining left/right hand with contralateral foot. It can be observed that not only the spatial distribution coefficient of left hand combined with contralateral foot imagery is significantly higher than that of left hand and feet imagery, but the spatial distribution coefficient of right hand combined with contralateral foot imagery is significantly higher than that of right hand and feet imagery as well.

**Figure 9:**
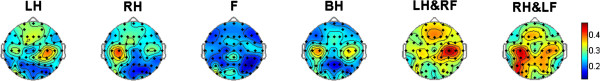
**The topographical distribution based on *q *values for 6 mental tasks.** Red regions indicate the activated areas during six types of motor imagery.

The topographical distributions based on the *q* values for 6 mental tasks are presented in Figure 9. It shows the relatively equalizing activation of bilateral hand areas during both hands imagery, which is different from the obvious contralateral dominance during single hand imagery. Although activated areas are mainly concentrated in the contralateral hand regions, more areas are activated during left/right hand combined with contralateral foot imagery, especially the frontal location and bilateral regions in the occipital location.

### Classification performance

For each subject, the CSP filters for each mental task were achieved on the training set. Then the log-variances of the spatially filtered EEG data were used as the extracted features. Moreover, for each direction of imagined movement, only the eigenvectors corresponding to first *k* eigenvalues could be used as spatial filters to extract features which are most suitable for classification. *k* = argmax(*acc*_CV_*(k))* is the number of spatial filters used in the classification with the highest average accuracy during tenfold cross-validation. To validate the separability of seven types of mental tasks, and compare three kinds of multi-class CSP algorithms in this study, we analyzed the classification accuracy by SVM. Table 1 shows the classification accuracies of ten subjects obtained on the test sets with highest accuracy of 84% and mean accuracy of 70%. From the results, we can see that the best multi-class CSP algorithm on data of seven mental tasks is Multi-sTRCSP with the highest mean accuracy. Taking a close look at the classification result, S2, S6 and S7 perform best under the Multi-sTRCSP approach with the accuracy above 75%. Both Multi-CSP and Multi-sTRCSP are significantly better than Multi-GECSP which is outperformed by about 2% in mean classification accuracy, at the 5% significance level using *t*-test (p = 0.0374 and 0.0078). However, Multi-sTRCSP preforms only slightly better than Multi-CSP, and there is no significant difference between each other.

**Table 1:** Classification accuracies of seven mental tasks for each subject

**Subject**	**S1**	**S2**	**S3**	**S4**	**S5**	**S6**	**S7**	**S8**	**S9**	**S10**	**mean**
Multi-CSP	70.82	81.79	**63.14**	64.28	**67.85**	74.11	71.61	**68.93**	71.96	**66.25**	70.07
Multi-GECSP	70.00	80.54	**63.14**	62.32	65.00	73.75	73.39	66.07	**72.50**	60.54	68.73
Multi-sTRCSP	**73.67**	**84.11**	62.07	**64.64**	66.07	**75.00**	**75.00**	68.75	71.07	63.93	**70.43**

Highest accuracy in each column is in bold font.

## Discussion

### Characteristics of compound limb motor imagery

From the result, a contralateral dominance is not observed during left hand imagery. The similar ERD pattern and spatial distribution during left hand motor imagery were also revealed by an investigation of four different MI tasks [17], which is probably due to the right handedness. The imagination of feet movement desynchronized the alpha band over not only the feet but also the hand representation area, which is similar to that revealed by ERD maps on a realistic head model during voluntary foot movement [32]. Movement imagination desynchronizes lower mu components somatotopically unspecific, which means desynchronization is present in all sensorimotor areas (in target attended and non-attended body part areas). But this widespread foot area ERD in alpha band was not found in every subject [17].

Mean power spectral density provides a more intuitive and convenient approach to observe the changes of ERD bands. Besides clear expansion of ERD band within alpha rhythm, beta band is also affected to some extent around 20Hz and even more high frequency components during compound limb motor imagery combining left/right hand with contralateral foot. However, the induced ERD mainly impacts on the narrow bands within beta rhythm during both hands imagery. As we know, the most reactive mu components of ERD with hand imagery are not exactly same as that with foot imagery [17], which means that the ERD band range of each other within alpha rhythm may exist deviation to some extent, so does the ERD band within beta rhythm probably. Meanwhile, the ERD components within alpha rhythm are different between voluntary hand and foot movement [32]. Therefore, the induced ERD may affect more broad-banded components within both alpha and beta rhythms at a certain degree during left/right hand combined with contralateral foot imagery.

In this study, PSE was used to evaluate the spectral distribution of EEG signals during imagination of different movements. A high PSE implies a uniform distribution spectrum of EEG signal within 5-35Hz, namely in terms of the ERD phenomenon studied here, more broad-banded ERD contributes to higher PSE values. As showed in Figures 3, 4 and 5, compared with simple limb motor imagery, more broad-banded ERD within both alpha and beta rhythms are observed during left/right hand combined with contralateral foot imagery. Correspondingly, the PSE values are indeed significantly higher than that of simple limb motor imagery in ten comparison groups at electrode positions C3, Cz and C4. Therefore, PSE can be regarded as a parameter to assess the ERD bandwidth in motor imagery paradigm.

Moreover, because of the simultaneous imagination of both hands, the bilateral hand areas are activated simultaneously. However, the situation, the ERD is slightly weaker in right hemisphere as compared to left hemisphere during both hands imagery, is probably attributed to the right handedness, namely more neurons have been activated in the right hand region. Besides, simultaneous imagination of upper limb and contralateral lower limb certainly contributes to the simultaneous activation of contralateral hand area and midcentral foot area, at the same time, the homolatertal hand area is also activated due to the influence on non-attended areas within lower mu components [17]. Such phenomenon implies the probability of the application of compound limb motor imagery to rehabilitation for the patients suffered from severe motor injury.

The topographical distribution from one individual shows us the existing differences on spatial distribution among seven mental tasks, additionally, spatial distribution coefficient was proposed for further investigation over all subjects. As showed in Figure 8, the spatial distribution coefficients of left/right hand combined with contralateral foot imagery are significantly higher than that of simple limb motor imagery, which means that the power distribution on the scalp is more uniform during left/right hand combined with contralateral foot imagery. Correspondingly, compound limb motor imagery combing left/right hand with contralateral foot indeed activate more function areas on cerebral cortex as well as sensorimotor areas. Therefore, spatial distribution coefficient can be regarded as a parameter to evaluate activation degree of the areas on cerebral cortex in motor imagery paradigm.

Phase synchronization study suggested that there probably existed a closer collaborative relationship between the SMA and M1 during the motor imagination combining body with limb action [11]. As mentioned above, due to the involvement of upper and lower limbs together, corresponding regions accompanied by neighboring cortical areas are activated simultaneously. So different function areas probably influence and cooperate with each other during the imagination of left/right hand combined with contralateral foot, which results in the changes on ERD bands and activated regions.

### Spatial patterns in CSP

**Figure 10:**
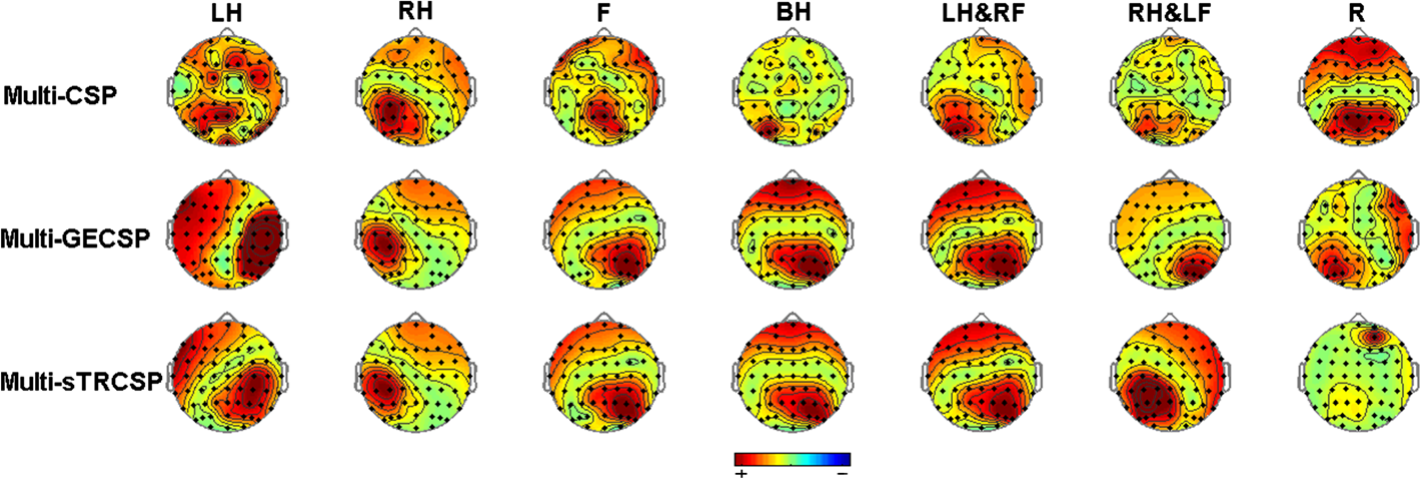
**The spatial patterns obtained with different multi-class CSP algorithms for one subject.** Topographical distributions are made based on the spatial patterns. High weight locations were represented by red for each task.

The spatial patterns of seven kinds of mental tasks obtained with different multi-class CSP algorithms are visualized in Figure 10, which can be used to verify the neurophysiological plausibility of ERD/ERS for different types of motor imagery [27]. We can see that the spatial patterns of Multi-CSP appear as messy such as left hand imagery, with large weight in unexpected electrode locations on brain surface. The location with high weight is opposite to the right hand representation area in spatial patterns of Multi-GECSP during right hand combined with left foot imagery. On the contrary, the Multi-sTRCSP spatial patterns are physiologically more relevant and neurophysiologically more plausible from a neurophysiological point of view.

In terms of the classification result, the modified multi-class CSP algorithms applied in this study are verified to be feasible to discriminate compound limb motor imagery among seven kinds of mental tasks. Indeed, the purpose of TRCSP algorithm is to generate better filters to diminish the influence of artifacts and avoid overfitting, otherwise sCSP is intended to guarantee stationary features [28]. In terms of the performance, benefiting from combining Tikhonov regularization and stationarity together, Multi-sTRCSP outperforms both Multi-CSP and Multi-GECSP. So multi-class motor imagery and the feature extraction method studied in this paper could be expected to provide technical support to expand the instructions of MI based BCI systems effectively.

## Conclusions

This study investigated the differences of the EEG patterns between three kinds of simple limb motor imagery and three kinds of compound limb motor imagery designed here by event-related spectral perturbation, power spectral entropy and spatial distribution coefficient. Moreover, three modified multi-class CSP algorithms were used to extract feature of seven mental tasks. The work implies that there exist the separable differences between simple limb motor imagery and compound limb motor imagery, which can be utilized to build a multimodal classification paradigm in motor imagery based BCI systems.

## Abbreviations

BCI: Brain-computer interface; ERD: Event-related desynchronization; ERS: Event-related synchronization; CSP: Common spatial patterns; Multi-CSP: Multi-class CSP; Multi-GECSP: Multi-class CSP based on generalized eigenvector; Multi-sTRCSP: Multi-class stationary Tikhonov regularized CSP; ERSP: Event-related spectral perturbation; PSE: Power spectral entropy; SVM: Support vector machine

## Competing interests

The authors declare that they have no competing interests.

## Authors' contributions

WY, DM and HQ designed the method and drafted the manuscript. WY and SQ carried out the data acquisition and analysis. LZ and BW supervised the study, helped revise the manuscript. All authors read and approved the final manuscript.
